# Specialist mental health crisis centres in England: a step forward or a stumble in the dark?

**DOI:** 10.1192/bjo.2025.10820

**Published:** 2025-08-19

**Authors:** Yasser Saeed Khan, Subodh Dave, Mohammed Al-Uzri, Javed Latoo, Ovais Wadoo

**Affiliations:** Royal College of Psychiatrists, London, UK; Hamad Medical Corporation, Doha, Qatar; Derbyshire Healthcare NHS Foundation Trust, Derby, UK; Leicestertershire Partnership NHS Trust, Leicester, UK; University of Bolton, UK; University of Leicester, UK; College of Medicine, Qatar University, Doha, Qatar

**Keywords:** Mental health, crisis, emergency, co-location, fragmented care

## Abstract

The recent proposal by NHS England to establish specialist mental health crisis centres has prompted considerable discussion. This editorial examines the initiative, which aims to reduce accident and emergency pressure and provide tailored care. However, it raises significant questions about the potential to exacerbate systemic fragmentation. Concerns highlight inadequate funding, the risk of resegregation of mental health from physical care and increased stigma if not properly integrated. This article argues that true holistic care requires seamless integration, advocating strongly for co-located mental health and medical emergency departments, which have shown improved outcomes. Ultimately, the success of these centres depends on addressing wider NHS issues, robust evaluation and a comprehensive vision prioritising the entire mental health pathway, from prevention to long-term recovery, to genuinely transform patient lives.

**Figure:**
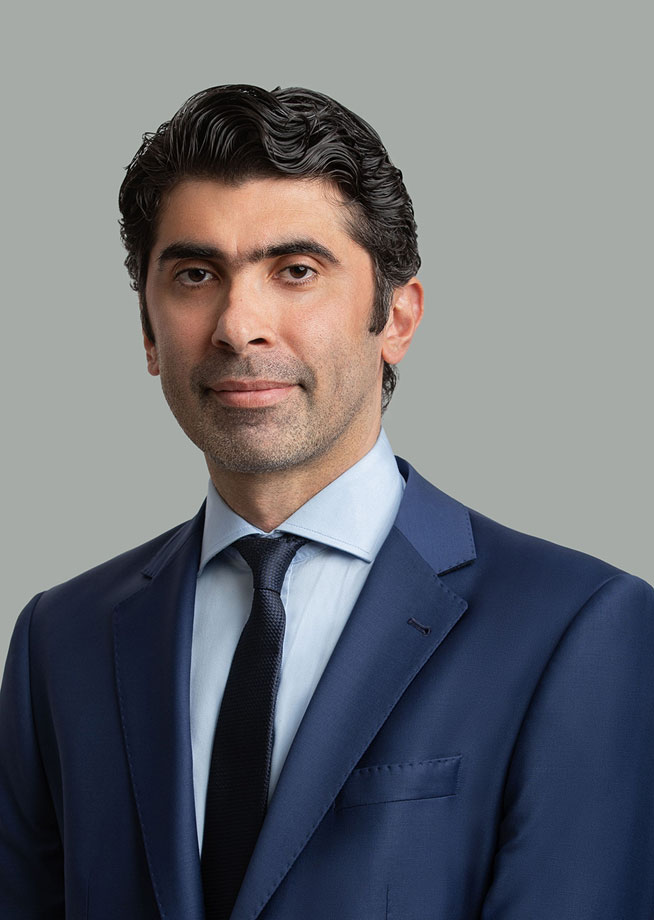

The recent announcement by National Health Service (NHS) England regarding the expansion of specialist mental health crisis centres across the country, aiming to alleviate pressure on overwhelmed accident and emergency (A&E) departments, presents a complex and timely discussion for the psychiatric community.^1^ While the stated intentions of providing ‘appropriate care in a calm environment’ and ‘speeding up access to appropriate care’ are laudable, the potential benefits and pitfalls of this initiative require scrutiny to determine whether this proposal represents genuine innovation or risks deepening systemic fragmentation. Our concerns, particularly its implications for stigma, the socialisation of psychiatry and the crucial integration of physical and mental health, have been echoed similarly by Dr Lade Smith CBE, President of the Royal College of Psychiatrists, in her response to the development.^2^

A recent systematic review and metasynthesis starkly highlights the shortcomings of current emergency department care for young people in mental health crisis.^3^ Their findings reveal that A&E is often perceived as unable to meet mental health needs, actively exacerbating patient distress through stressful environments and long waits. Crucially, these adverse experiences lead to deterrence from future help-seeking and increased risk of subsequent self-harm or suicide. This evidence reinforces the urgent necessity for new models of care that are truly compassionate, person-centred and prevent the resegregation of mental health from integrated physical care. A qualitative analysis on psychosocial assessments following self-harm revealed that, while compassionate and supportive assessments were beneficial, negative experiences, stigmatising comments and even refusal of medical care were common. This study highlights that, even within mental health assessments, preconceived notions about self-harm as a ‘behavioural issue’ by some staff can lead to greater distress and disengagement, directly compounding concerns about stigma and the quality of care.^4^

It is recognised that a detrimental cycle can emerge from negative A&E encounters.^5^ These experiences can deepen a patient’s negative self-perception, leading to increased shame and a greater likelihood of avoiding services. The consequent reluctance to seek help, in turn, perpetuates the cycle of self-harm, a critical concern due to the elevated risk of recurrent self-harm and suicide.^6^

## The promise of dedicated crisis care

On the surface, redirecting individuals experiencing a mental health crisis away from overstretched A&E departments appears to be a pragmatic solution to a pervasive problem. The grim statistics of extended A&E waits, with over 60 000 individuals enduring waits of 12 h and more in January alone,^1^ highlight the urgent need for alternative pathways to care. Recent reports by the Royal College of Emergency Medicine^7^ have consistently shown that patients presenting with mental health problems are twice as likely to spend 12 h or more in emergency departments than other patients, often in environments unsuitable to managing their distress. A UK Parliament committee report^8^ further details the detrimental effects of A&E crowding, including increased mortality rates for patients experiencing delays beyond 5–6 h. These prolonged waits often signify a failure of initial triage and a broader systemic inability to anticipate and mitigate the complications of delayed care for mental health crises.

Dedicated mental health crisis centres, staffed by specialists and open to walk-in patients, as well as GP and police referrals, offer the promise of immediate, tailored support in an environment more conducive to addressing mental distress than the chaotic milieu of an emergency department. This ‘pioneering new model of care’, as Sir Jim Mackey, NHS England chief, describes it, could reduce the risk of further distress for vulnerable individuals and allow A&E staff to focus on acute physical emergencies. Furthermore, the very existence of such centres could serve to destigmatise mental health crises by acknowledging them as distinct and requiring specialised attention rather than being considered as an adjunct to physical illness. Clinical studies on crisis stabilisation centres in other contexts, such as the USA, show their effectiveness in suicide prevention, providing behavioural health treatment and diverting individuals from higher levels of care. Additionally, they contribute to reduced emergency department length of stay.^9^

## The risk of stigma, resegregation and neglect of holistic care

While the promise of dedicated crisis care is significant, there is a distinct risk that, by creating separate ‘mental health A&Es’, we further isolate mental health from physical health. This could implicitly reinforce a perception of mental illness as ‘other’ or less legitimate than physical ailments, undermining decades of effort to integrate mental health into mainstream medicine. A mixed-methods review found that service users consistently report poor experiences in A&E during mental health crises, arising from stigmatising staff attitudes and inadequate skills in managing mental health needs.^10^ It identifies stigma as an overarching social construct profoundly impacting care provision and service users’ emotional responses. This results in care being perceived as ineffective and increases patient distress. Positive experiences were largely attributed only to the presence of specialist mental health liaison services, highlighting the critical gaps in general A&E mental healthcare.

This leads directly to the concern about the socialisation of psychiatry into isolated settings, potentially reversing the progress made in integrating psychiatric understanding within general healthcare. It is impossible to separate out physical and mental health problems so simply. Many mental health crises are intricately linked to physical health conditions, substance misuse or medication side effects. The Royal College of Psychiatrists reinforces this, noting that estimates from the COVID-19 pandemic suggest between two-thirds and three-quarters of patients in acute mental health crisis present with coexisting physical conditions, and a significant 10% initially thought to have purely mental health needs are found to have a serious physical problem requiring transfer to emergency physical care.^2^

Individuals with severe mental illness also have higher rates of chronic medical conditions compared with the general population.^11,12^ Diverting individuals solely to mental health crisis centres without robust and immediate pathways for physical health assessment and intervention could lead to neglect of critical medical needs, a phenomenon known as diagnostic overshadowing.^13^ This bias, where symptoms of one illness are misattributed to a previousy diagnosed comorbidity, particularly mental illness, can lead to compromised patient care and contributes to the increased mortality experienced by individuals with mental illness.^13^

Furthermore, by removing mental health presentations from general acute settings, there is a risk of reducing the exposure of acute medical setting staff to the complex interplay of mental and physical health issues. This could inadvertently lead to a decrease in the general healthcare workforce’s overall competency and confidence in managing mental health crises, exacerbating diagnostic overshadowing and potentially leading to poorer physical health outcomes for individuals with mental health conditions. Having psychiatrists and psychiatric nurses embedded within acute medical settings is crucial because they can advocate for better, integrated physical healthcare for their patients.

Real-world clinical scenarios illustrate the danger of a siloed approach; patients with undiagnosed delirium, epilepsy or encephalitis may be misdirected to psychiatric services, delaying critical medical treatment. This siloed approach risks perpetuating the harmful mind–body dichotomy that psychiatry has long sought to overcome. True holistic care necessitates seamless integration, where behavioural health clinicians are embedded within general medical settings and vice versa.^14^ Evidence-based integrated care models, including the mental health integrated care model in Australia, have demonstrated benefits, highlighting factors such as co-location of services, multidisciplinary teams, shared protocols and consolidated physical and psychiatric clinical records.^15,16^ Apart from stigma, separation of mental and physical illness may lead to poorer overall health outcomes for people with long-term medical conditions. For instance, in chronic pain, diabetes and cardiovascular disease, psychiatric comorbidity is common and significantly impacts overall health, often leading to poorer care when unaddressed.^17^

Co-locating specialised mental health A&Es with general A&E departments has proven effective, notably in reducing waiting times for mental health patients, as demonstrated at Hull University Hospital.^2^ Other hospitals, including St Thomas’ Hospital and Barts Health, have also adapted their emergency departments to create designated spaces for people in mental health crisis. Conversely, creating stand-alone mental health facilities raises concerns about communication breakdowns, barriers and delays necessitating patient transfers, often by ambulance, between physical and mental health services. Lessons from past experiences, including during COVID-19, stress the critical need for co-located or linked services. Moreover, investing in evidence-based interventions such as liaison psychiatry services, which integrate hospital care for patients with co-occurring mental and physical illnesses, can significantly improve emergency care and lead to better patient outcomes.^2^

## A symptom of systemic underfunding?

The notion of expanding ‘dozens of locations’ with a £26 million investment, while a positive step, immediately raises questions about the long-term sustainability and adequacy of resources. From an economic perspective, a more robust argument for financial parity is needed. Will the proposed crisis centres be funded at the same level as general A&E departments, considering the complexity and duration of care often required for mental health crises? A cost–benefit analysis that considers the long-term savings from averted hospital admissions, reduced A&E burden and improved patient outcomes would provide a stronger economic justification. Without such parity and a clear return on investment model, these centres risk becoming under-resourced, exacerbating existing inequalities in healthcare provision.

The British Medical Association highlights that mental health funding, while increasing in cash terms, has not kept pace with overall NHS expenditure, and the proportion of the NHS England budget spent on mental health has actually fallen since 2016/17.^18^ This suggests that the proposed crisis centres may be less of a strategic enhancement and more of a manifestation of systemic underfunding within the broader mental health landscape. Inadequate funding could lead to understaffed centres, long waiting times within these new facilities themselves and, ultimately, failure to deliver on the promise of timely, quality care. This would not only undermine the initiative but could also erode public trust in mental health services, which is already significantly strained. This is evident from the 345 000 referrals waiting more than 1 year for first contact with mental health services as of June 2024, including 109 000 children and young people under the age of 18 years.^19^ Notably, mental illness has been excluded from NHS England’s elective waiting list initiatives.^20^

Moreover, the very concept of ‘crisis centres’ can, despite best intentions, reinforce perceptions of mental illness as an acute, episodic phenomenon rather than a chronic condition requiring ongoing integrated care. This could detract from the crucial need for robust preventative services, early intervention and long-term community support that are fundamental to preventing crises from originating. Recent analyses highlight that, while demand for children and young people’s mental health services has surged, NHS services struggle to meet this need, with significant gaps in early support and increasing numbers reaching crisis point.^21^ Clinical guidelines advocate for a comprehensive crisis care approach, including linkage to ongoing support and addressing underlying social determinants of health.^22^

## Future directions

The proposed expansion, while offering a much-needed alternative to A&E, demands rigorous evaluation and continuous refinement. The untrialled nature of the model demands robust testing before widespread national rollout. Evaluation must include not only clinical outcomes but also assessment of integration with primary care, acute physical healthcare and community mental health services. Crucially, the development of any new model should strongly consider the benefits of co-located mental health and acute medical emergency departments.

The success of any new model of care will also depend on addressing wider NHS crises, such as staffing shortages and burnout among healthcare workers. It is also crucial to recognise that long waiting times in A&Es have been a symptom of significant bed reductions over the past couple of decades. The lack of appropriate step-down accommodation for psychiatric in-patients directly contributes to the long waits, a complex issue that simply won’t be resolved by establishing separate mental health crisis centres.

In conclusion, while the establishment of specialist mental health crisis centres may offer a promising alternative, it requires a strong commitment to seamless integration with physical healthcare. The co-location of mental health and acute medical A&Es, as proposed by the Royal College of Psychiatrists, can avoid resegregation and ensure holistic care. Furthermore, investing in 24-h crisis teams could serve as a potential alternative for individuals known to services, providing continuity of care and potentially preventing crises from escalating to emergency levels. Finally, a comprehensive vision must prioritise the entire mental health pathway, from prevention to long-term recovery. Without these considerations, this initiative risks becoming a well-intentioned but inadequate attempt to address a complex societal challenge, potentially deepening stigma and exacerbating fragmentation. As psychiatrists we must advocate not just for more services but for better, integrated and truly holistic care that genuinely transforms the lives of those living with mental illness.

## Data Availability

Data availability is not applicable to this article because no new data were created or analysed.
